# Supplementary material for: What emotions does music express? Structure of affect terms in music using iterative crowdsourcing paradigm
Source: PLoS One. 2025 Jan 22;20(1):e0313502. doi: 10.1371/journal.pone.0313502 (PMC11753638; doi:10.1371/journal.pone.0313502)
Supplement: S1 File — (PDF) [file pone.0313502.s001.pdf]

# Supporting Information

**S1 Demographic measures** General demographic information consisted of

1. **Musical expertise*** on a self-reported scale from *1=nonmusician – 7=professional musician*

2. **Language skill** on a scale from *1=no proficiency – 6=native or bilingual proficiency*

3. **Gender**

4. **Age**

5. **The country of the participant**, estimated based on the IP-address from where they participated.

**SI Table 1 Continents and countries of the participants in Experiment 1.**

| Continent | n | Country with most participants |
|---|---|---|
| Asia | 1607 | India, n = 297 |
| Europe | 979 | Bosnia and Herzegovina, n = 203 |
| South America | 510 | Venezuela, n = 277 |
| North America | 315 | United States, n = 136 |
| Africa | 80 | Egypt, n = 23 |
| Oceania | 10 | New Zealand, n = 6 |

Musical genre preferences were collected using a genre label checklist, organised in a hierarchy of 16 main genres with 7 subgenres each. Selecting a specific main genre opened a pop-up checklist of its subgenres. The main genres were adopted from the STOMP genre questionnaire [?], replacing "rap" and "heavy metal" with more general terms "hip hop" and "metal", respectively (based on the popularity of the usage of these tags in *Last.fm*). Moreover, genres "latin" and "reggae" were added as main genres to cover a wider variety of music. The subgenres were determined on the basis of cluster analysis of a large collection of genre tags from Last.fm tags, where the closest genre tags were associated with the main genres based on a computational semantic similarity measure. Seven of the most popular subgenres were retained for each main genre.

**SI Table 2 Percentage of participants liking each main genre in Experiment 1.**

| Genre | Participants (%) |
|---|---|
| Alternative | 22.63 |
| Blues | 18.46 |
| Classical | 30.54 |
| Country | 17.72 |
| Electronic | 27.80 |
| Folk | 14.27 |
| Hip Hop | 25.57 |
| Jazz | 18.41 |
| Latin | 17.72 |
| Metal | 23.14 |
| Pop | 55.28 |
| Reggae | 16.84 |
| Religious | 8.82 |
| Rock | 54.74 |
| Soul | 15.18 |
| Soundtrack | 23.34 |

**SI Table 3 Activity importance of the participants in general and while listening to music in Experiment 1.**

| Activity | General | Musical |
|---|---|---|
| | Mean (SD) | Mean (SD) |
| Daily routines | 3.40 (1.10) | 3.40 (1.15) |
| Emotional | 3.28 (1.15) | 3.40 (1.21) |
| Entertainment | 3.85 (1.02) | 3.62 (1.12) |
| Intellectual | 3.59 (1.14) | 2.93 (1.27) |
| Live music | 3.14 (1.22) | 3.73 (1.23) |
| Music listening | 3.87 (1.03) | 4.17 (1.02) |
| On the move | 3.73 (1.04) | 3.79 (1.11) |
| Physical | 3.48 (1.09) | 3.75 (1.11) |
| Social | 3.26 (1.10) | 3.40 (1.14) |

## SI Table 4 Prevalence of affect terms in Experiment 2.

| Term | Rank | Freq | F | Term | Rank | Freq | F |
|---|---|---|---|---|---|---|---|
| adorable | 64 | 0.079 | 0.065 | **jumpy** | 38 | 0.107 | 0.067 |
| *aggressive* | 52 | 0.090 | 0.063 | liberated | 56 | 0.090 | 0.073 |
| amazed | 76 | 0.071 | 0.062 | light-hearted | 57 | 0.094 | 0.071 |
| **angry** | 81 | 0.057 | 0.060 | *liked* | 49 | 0.104 | 0.069 |
| animated | 65 | 0.083 | 0.058 | **loud** | 39 | 0.112 | 0.057 |
| bitchy | 85 | 0.050 | 0.065 | **melancholic** | 40 | 0.111 | 0.064 |
| bored | 77 | 0.072 | 0.064 | moody | 50 | 0.103 | 0.056 |
| *celebratory* | 53 | 0.091 | 0.071 | *motivational* | 41 | 0.111 | 0.066 |
| *cool* | 5 | 0.175 | 0.066 | nocturnal | 70 | 0.078 | 0.062 |
| **dancing** | 3 | 0.182 | 0.068 | *passionate* | 12 | 0.152 | 0.061 |
| *deep* | 34 | 0.107 | 0.073 | *peaceful* | 24 | 0.128 | 0.069 |
| delighted | 66 | 0.084 | 0.066 | **playful** | 19 | 0.142 | 0.060 |
| *dramatic* | 44 | 0.099 | 0.064 | *pleasant* | 13 | 0.148 | 0.072 |
| *dreamy* | 20 | 0.127 | 0.065 | *positive* | 8 | 0.164 | 0.065 |
| *driving* | 54 | 0.094 | 0.062 | **powerful** | 14 | 0.146 | 0.067 |
| *easy* | 27 | 0.118 | 0.057 | pulsating | 30 | 0.122 | 0.064 |
| elegant | 45 | 0.100 | 0.063 | reflective | 71 | 0.085 | 0.069 |
| *emotional* | 4 | 0.178 | 0.065 | *relaxed* | 9 | 0.164 | 0.068 |
| encouraged | 78 | 0.069 | 0.058 | religious | 87 | 0.039 | 0.071 |
| **energetic** | 2 | 0.206 | 0.066 | repetitive | 58 | 0.085 | 0.061 |
| *enthusiastic* | 21 | 0.128 | 0.068 | retro | 42 | 0.105 | 0.063 |
| epic | 79 | 0.073 | 0.064 | *rhythmical* | 1 | 0.220 | 0.069 |
| *euphoric* | 35 | 0.108 | 0.063 | **romantic** | 10 | 0.157 | 0.064 |
| **excited** | 16 | 0.137 | 0.066 | **sad** | 72 | 0.077 | 0.065 |
| fantastic | 46 | 0.097 | 0.069 | satisfied | 73 | 0.079 | 0.053 |
| **fast** | 36 | 0.108 | 0.069 | *sensual* | 31 | 0.121 | 0.063 |
| *festive* | 47 | 0.103 | 0.067 | *sentimental* | 25 | 0.134 | 0.069 |
| *free* | 28 | 0.117 | 0.060 | serene | 74 | 0.080 | 0.067 |
| **friendly** | 29 | 0.124 | 0.071 | severe | 86 | 0.045 | 0.061 |
| **fun** | 17 | 0.136 | 0.065 | *sexual* | 83 | 0.061 | 0.065 |
| *funny* | 55 | 0.087 | 0.065 | *sexy* | 59 | 0.085 | 0.062 |
| **furious** | 67 | 0.075 | 0.072 | **slow** | 26 | 0.131 | 0.066 |
| gentle | 37 | 0.111 | 0.068 | **smooth** | 32 | 0.116 | 0.064 |
| glorious | 68 | 0.079 | 0.067 | **soft** | 15 | 0.151 | 0.070 |
| **good** | 6 | 0.165 | 0.061 | sorry | 88 | 0.042 | 0.070 |
| graceful | 48 | 0.097 | 0.073 | *spiritual* | 60 | 0.087 | 0.067 |
| *happy* | 11 | 0.150 | 0.066 | spontaneous | 61 | 0.088 | 0.071 |
| *heroic* | 82 | 0.063 | 0.068 | stimulated | 33 | 0.117 | 0.066 |
| *hot* | 80 | 0.071 | 0.071 | **strong** | 43 | 0.114 | 0.065 |
| **in love** | 18 | 0.140 | 0.063 | stylish | 51 | 0.101 | 0.053 |
| *inspired* | 22 | 0.130 | 0.066 | *successful* | 75 | 0.085 | 0.061 |
| **intense** | 23 | 0.134 | 0.072 | tragic | 84 | 0.059 | 0.066 |
| interested | 69 | 0.084 | 0.062 | tranquil | 62 | 0.095 | 0.059 |
| **joyful** | 7 | 0.164 | 0.070 | uplifting | 63 | 0.094 | 0.062 |

*Rank of frequency* and *frequency* of mood terms for identified tracks, *F* for F-measure of terms retrieved in track annotation task, and set of *55* and **23** terms.

## SI Table 5 Affect terms present in each model of Experiment 2.

| Term | Original | Alpha | Combined | Optimal | Manual | MIREX | GEMS | GEMIAC |
|---|---|---|---|---|---|---|---|---|
| aggressive | F1 | | | | M1 | | TENSION | G14 |
| angry | F2 | A1 | C1 | O1 | M1 | | | G13 |
| celebratory | F3 | A2 | C2 | | M2 | | | |
| cool | F4 | | | | M3 | | | |
| dancing | F5 | A3 | C3 | O2 | M4 | | JOYFUL | G6 |
| deep | F6 | | | | M5 | | WONDER | G12 |
| dramatic | F7 | A4 | C4 | | M6 | | WONDER | G1 |
| dreamy | F8 | | | | M5 | MIREX1 | | G9 |
| driving | F9 | | | | | | | |
| easy | F6 | A6 | C5 | | | | | |
| emotional | F10 | | | | M7 | MIREX1 | NOSTALG. | G2 |
| energetic | F5 | A3 | C3 | O2 | M4 | MIREX2 | POWER | G5 |
| enthusiastic | F9 | | | | | | | G4 |
| euphoric | F11 | A5 | C3 | | | | TRANSC. | G3 |
| excited | F6 | A6 | C3 | O2 | | MIREX2 | | G5 |
| fast | F7 | A4 | C3 | O2 | M4 | | | |
| festive | F3 | | | | M2 | | | |
| free | F6 | A6 | C5 | | M3 | | | |
| friendly | F12 | A7 | C6 | O3 | M8 | | | |
| fun | F13 | A8 | C2 | O4 | M9 | MIREX3 | | |
| funny | F8 | | | | M9 | MIREX4 | | |
| furious | F1 | A9 | C1 | O1 | M1 | | | G14 |
| good | F12 | A7 | C6 | O3 | M3 | | | |
| happy | F14 | | | | M9 | MIREX3 | JOYFUL | G6 |
| heroic | F15 | A10 | C7 | | M10 | | POWER | G1 |
| hot | F16 | | | | M11 | | | |
| in love | F10 | A11 | C8 | O5 | M12 | | TENDERN. | G8 |
| inspired | F16 | | | | M10 | | WONDER | G4 |
| intense | F17 | A12 | C1 | O1 | | MIREX5 | | G2 |
| joyful | F13 | A8 | C2 | O4 | M2 | MIREX3 | JOYFUL | G6 |
| jumpy | F18 | A13 | C3 | O2 | M4 | MIREX5 | TENSION | G13 |
| liked | F12 | A7 | C6 | | M8 | | | |
| loud | F17 | A12 | C1 | O1 | | | | |
| melancholic | F19 | A14 | C9 | O6 | M6 | MIREX1 | SADNESS | G10 |
| motivational | F15 | A10 | C7 | | M10 | MIREX5 | | |
| passionate | F6 | | | | M7 | | | |
| peaceful | F8 | | | | M13 | | PEACEF. | G9 |
| playful | F13 | A8 | C2 | O4 | | MIREX4 | | |
| pleasant | F8 | | | | | | | |
| positive | F1 | | | | | | | |
| powerful | F17 | A12 | C1 | O1 | M14 | | | G7 |
| relaxed | F8 | | | | M13 | | PEACEF. | G9 |
| rhythmical | F5 | A3 | C3 | | M4 | | | |
| romantic | F10 | A11 | C8 | O5 | M12 | | | G11 |
| sad | F19 | A14 | C9 | O6 | M6 | | SADNESS | G10 |
| sensual | F14 | A15 | C10 | | M11 | | | |
| sentimental | F10 | | | | M12 | MIREX1 | NOSTALG. | G11 |
| sexual | F4 | A16 | C11 | | M11 | | | |
| sexy | F8 | | | | M11 | | | |
| slow | F5 | A3 | C9 | O6 | M5 | | | G12 |
| smooth | F20 | A17 | C5 | O7 | M13 | | PEACEF. | |
| soft | F2 | A1 | C5 | O7 | M13 | | TENDERN. | G8 |
| spiritual | F21 | A18 | C12 | | | MIREX1 | TRANSC. | G3 |
| strong | F17 | A12 | C1 | O1 | M14 | MIREX2 | POWER | G7 |
| successful | F15 | A10 | C7 | | | | | |
| Items | 36 | 36 | 43 | 16 | 22 | 30 | | |
| Factors | 21 | 18 | 12 | 7 | 14 | 5 | 9 | 14 |

**SI Original terms** abstract, accepted, acerbic, active, admired, adorable, adventurous, affectionate, afraid, aggravated, aggressive, agitated, agonised, agreeable, airy, alarmed, alienated, allured, amazed, ambitious, amiable, amorous, amused, angry, angst-ridden, anguished, angular, animated, annoyed, anticipated, anxious, apathetic, apocalyptic, apprehensive, arid, aroused, astonished, athletic, atmospheric, attentive, attractive, austere, autumnal, beautiful, bellicose, belligerent, benevolent, bitchy, bitter, bittersweet, bizarre, bleak, blissful, blustered, boisterous, bold, bombastic, booming, bored, bouncy, brash, brassy, bravado, brave, bright, brittle, brooding, building, buoyant, busy, calm, campy, capricious, caring, carefree, careful, caressing, cartoonish, cathartic, cautious, celebratory, cerebral, certain, chaotic, cheeky, cheerful, child-like, childish, circular, climactic, clinical, close, cold, comforting, comic, compassionate, complex, conceited, concerned, concise, confident, confrontational, confused, conscientious, consoling, contemplative, contemptuous, content, convincible, cool, cosmopolitan, courageous, crazy, creepy, crunchy, crunk, curious, cynical, dancing, dangerous, dark, dazzled, declamatory, deep, defeated, defiant, dejected, delicate, delighted, demonic, depressed, desired, despaired, despondent, detached, determined, devotional, difficult, dignified, disappointed, discontent, disenchanted, disgusted, disillusioned, disinhibited, disliked, dismayed, displeased, disregarded, dissatisfied, dissonant, distraught, distressed, distrusted, disturbed, doleful, doubtful, dramatic, dreadful, dreamy, driving, droning, droopy, druggy, drunk, dynamic, eager, earnest, earthy, easy, ebullient, eccentric, ecstatic, edgy, eerie, effervescent, elaborated, elated, electrified, elegant, elegiac, embarrassed, emotional, empathic, emphatic, enchanted, encouraged, energetic, enigmatic, enjoyed, enraged, enthralled, enthusiastic, envious, epic, erotic, escalated, ethereal, euphoric, evil, exasperated, excited, exhausted, exhilarated, exotic, expectant, explosive, extrovert, exuberant, faithful, fancied, fantastic, fantasy-like, fast, fatigue, fearful, feral, ferocious, fervor , festive, feverish, fierce, fiery, flashy, flirty, floating, flowing, fond, forceful, foreboding, forgiving, fractured, frantic, freaky, free, freewheeling, fresh, friendly, frightened, frisky, frustrated, fun, funereal, funky, funny, furious, gay, gentle, giddy, glad, gleeful, gloomy, glorious, glum, good, good-natured, goose bumps, graceful, grand, greasy, grieving, grim, gritty, groovy, grouchy, grumpy, guilty, gutsy, happy, hard, harsh, hateful, haunting, heartbroken, heartening, heartwarming, heated, heavenly, heavy, hectic, hedonistic, helpless, heroic, hesitant, homesick, honest, honour, hopeful, hopeless, horny, horrified, hostile, hot, humble, humiliated, humorous, hungry, hurt, hymn-like, hyper, hypnotic, hysteric, impatient, impressed, improvisatory, in love, indifferent, indignant, indulgent, infatuated, innocent, inquisitive, insecure, inspired, insular, insulted, intelligent, intense, interested, intimate, intricate, introspective, ironic, irreverent, irritated, isolated, jangly, jealous, jittery, jolly, jovial, joyful, jubilated, jumpy, kind, kinetic, knotty, laid-back, languid, languorous, lazy, liberated, light, light-hearted, liked, literate, lively, loathed, lofty, lonely, longing, lost, loud, loved, low-spirited, lush, lusted, lyrical, macabre, mad, magical, majestic, malevolent, manic, marching, martial, mean, meandering, meaningful, mechanical, meditative, melancholic, mellow, menacing, messy, mighty, mischievous, miserable, monastic, monumental, moody, mortified, motivational, motoric, mournful, moved, mysterious, mystical, naive, narcotic, narrative, negative, neglected, nervous, nihilistic, noble, nocturnal, nonchalant, nostalgic, obsessive, ominous, oppressed, optimistic, opulent, organic, ornate, outgoing, outraged, outrageous, painful, panicky, paranoid, passionate, passive, pastoral, patriotic, peaceful, pensive, perky, philosophical, pious, piqued, pissed off, pity, plain, plaintive, playful, pleading, pleasant, poignant, polite, pompous, positive, powerful, precious, proud, primitive, provocative, pulsating, pulsing, pure, purposeful, pushy, questioning, quiet, quirky, raging, rambling, rambunctious, ramshackle, raptured, raucous, raunchy, raw, reassuring, rebellious, reckless, refined, reflective, regal, regretful, rejected, relaxed, relieved, religious, relived, remorseful, repetitive, resentive, reserved, resolute, respected, restless, restrained, retro, reverent, revolted, revulsed, rhapsodic, rhythmical, risque, rollicking, romantic, rousing, rowdy, rustic, sacred, sad, sarcastic, sardonic, satirical, satisfied, savage, scary, scattered, schmaltzy, scornful, searching, secretive, sedate, self-confident, self-conscious, sensitive, sensual, sentimental, serene, serious, severe, sexual, sexy, shameful, shimmering, shivery, shocked, sick, silly, simple, sincere, sinister, sleazy, sleepy, slick, slow, smoky, smooth, sneaky, snide, snobbish, soaring, soft, solemn, somber, soothing, sophisticated, sorrowful, sorry, soulful, spacey, spacious, sparkling, sparse, spicy, spirited, spiritual, spited, spontaneous, spooky, sprawling, sprightly, springlike, stable, startled, stately, stimulated, stirring, strange, street-smart, strong, striding, striking, stylish, sublime, subtle, successful, suffered, suffocating, sugary, suggestive, summery, superior, surprised, suspenseful, suspicious, swaggering, sweeping, sweet, swinging, swirling, sympathetic, taken aback, technical, tender, tense, terrified, thankful, theatrical, thoughtful, threatening, thrilling, thuggish, tired, tormented, touched, tough, tragic, trancelike, tranquil, transcendent, translucent, transparent, trashy, trippy, triumphant, trustful, tuneful, turbulent, ugly, uncertain, uncomfortable, uncompromising, understated, uneasy, unfortunate, unfriendly, unhappy, unpleasant, unsettled, uplifting, urgent, vengeful, vibrant, virile, visceral, volatile, voluptuous, vulgar, warm, wavering, weak, weary, well, whimsical, wholesome, wicked, wild, wintry, wistful, witty, woeful, wondrous, worried, wrathful, wrong, wry, yearning, zealless, zestful
