# Supplementary material for: What emotions does music express? Structure of affect terms in music using iterative crowdsourcing paradigm
Source: PLoS One. 2025 Jan 22;20(1):e0313502. doi: 10.1371/journal.pone.0313502 (PMC11753638; doi:10.1371/journal.pone.0313502)
Supplement: Attachment — Submitted filename: PlosOne emotions expressed in music.pdf [file pone.0313502.s002.pdf]

This is a very interesting and rigorously presented research study on emotions associated with music. The statistical analyses are well documented and seem to be done properly, but I am no expert so I cannot confirm this with full confidence.

My main concerns and request for revision are the following:

1) The methodology employed in the research is quite different from previous work that aimed at finding the main emotion terms that can be associated with music. It is not clear how deliberate this is. Are the authors arguing that this way of identifying emotion terms is more valid than previous surveys? If so, this should be part of the discussion of previous approaches in the literature review and introduction. Or was the study originally conceived to serve a different purpose (more about the emotional functions of music in everyday life) and was (part of) the data collected used for the purpose of identifying expressed emotions in music? If so, this should be acknowledged more openly.

2) Related to point 1, I feel there should be a more transparent discussion of the strengths and weaknesses of the approach taken in this study in the discussion. The way these terms were chosen, are they strictly speaking about what emotions music can express or are they indicating the affects and emotions that music can afford to listeners in certain circumstances? Such a relational perspective can be seen as a strength of the approach. It is about what music affords to listeners rather than purely about the potential communicative power of music and its characteristics. The role of lyrics in this sample of music should also be acknowledged.

3) More generally what are the limitations that the approach taken may have promoted? The terms are chosen first of all in relation to activities that they can be associated with, and only quite a small number of activities are included. For example, mediation, sleep or connecting spiritually are not included, nor are having sex or preparing for war. A larger range of activities could have shown a larger range of expressions?

4) The literature review is not exhaustive. Whilst that may not be the purpose of the literature review in this case, the writing does suggest as if all relevant most recent literature has been included. However that is not the case – the below is for example also a relevant article. Also, did not GEMS also include expressed emotion? The 11 terms used in Laukka et al. cross-cultural study (2013, Emotion) seems also relevant?

Schubert, E. (2003). Update of the Hevner adjective checklist. *Perceptual and motor skills*, *96*(3_suppl), 1117-1122.

5) A discussion of the terms is important too – affect terms, emotions, or even more broadly? The introduction is formulated in a way that suggests that this research will give an updated and more definitive list of emotions terms expressed by music. These promises should be checked with what the study actually offers in the end including in light of its limitations.

6) Abstract also needs to be updated in light of these comments.

5) The English is not yet good enough. There are a lot of awkward sentences with poor grammar, which makes the reading challenging and reduces the clarity of what is written.

Minor comments:

P2/30 l42. ‘inferior model’. This seems a bit strongly formulated. Also other models? How many, which do you mean?

P3/30 l71. Some missing references such as to Hevner and Schubert.

Figure 1 legend – Level a refers to the input ‘of’ or input ‘to’? I think it should be ‘to’.

L127/8. Rather abstract first sentence under Rationale. Please improve.

L141. You regularly state ‘aim’, which makes it confusing what the aim of the research is.

L150. Why is it important to collect this contextual information. This is buried in the methodology and needs more explicit discussion (see general comments).

L162. Limited working proficiency? What is meant by that? Are you talking about English language proficiency?

L169. Western music and classical music -> clarify this further, be more precise.

L171. You present various statistics here but have not yet explained that you would be collecting those. Add a signposting explanation before summarising the data.

L225. Low ability to associate tracks – could it be that some search terms are less successful in retrieving an appropriate track, and could this be a confounding factor?

L243 Silhouette scores? More info is needed.

L247 not clear what analysis was done to reduce to 88 clusters. There were 9 activities, so how did those play a role in the reduction as suggested above?

Figure 2. Panel A, B and C are not matched in order with the order presented in the figure caption.

L264-5. Check grammar

L282 and elsewhere. Check what tense you use where. In Exp 2, method, you used past tense. Here you use present. (aim was / aim is).

L302. do you mean that participants did two times an activity association task and four times an annotation subtask? They could not do as many tasks anymore as they wanted? The way this is phrased is unclear as it can also be two different activity association tasks.

L307 and the rest of that section. This is data analysis, not data collection procedure.

L314. This procedure (division) is that to get a proportion? Can be further explained.

L322. Why binary terms and why 22. I am lost a bit here without further explanation.

L330. Not majority of the track, but the largest number of tracks?

L333. The median of 211 activities associations were obtained. This is phrased in a rather confusing manner. Please rephrase and clarify. Also, 211 unique associations or are just reporting how many responses? I assume the latter. But that is not so relevant, is it?

L373. ‘separated into two’. Do you mean each was split into to two to yield 10, or combined into two factors?

L374. by considering computing? Or do you mean by computing? Surely you do not just consider it?

L375. were attempted to combine is not good grammar

L421. In General Discussion. ‘the’ is missing.

L433. Did participants clearly chose music to express this? I would discuss and unpick this a bit further (see general comments).

L443. Further data – as will be done next.

L445-7. First sentence of the section is hard to parse. Please improve.

L479-480. check sentence the factors are collected in this experiment, are not they? Or perhaps 'with' is not the correct word?

Table 3. table heading. not clear what is being correlated as you say 2x 'between' in this sentence.

Table 3 and elsewhere: check when you do and do not include a 0 before the full stop. You say $p < .01$ but also $r = 0.01$. All numbers that range between 0 and 1 should be treated the same, so this would need to be $r = .01$ to be consistent. (and making the r and p italic).

L497. Dreamy is ‘simply’. Why simply and how can you be so sure?

L499. what do you mean by 'the more dimensional concepts?

L502. and inspired and sensual are themselves the most poorly defined?
L528 and elsewhere. affect terms or concepts? You switched terminology. Be consistent for clarity. Or is this change on purpose?

L534. acknowledge that quadrant is close to .9

L605. different sets of structures -> is this undermining the confidence in the outcomes too much? Formulate less strongly?

L625. ‘less descriptive’ should this be ‘fewer descriptive’?

S1 Table 1. Why this order of continents? It is not alphabetical nor in order of number of participants.

S1 Table 4. How can the rank be alphabetical?
