# Supplementary material for: What emotions does music express? Structure of affect terms in music using iterative crowdsourcing paradigm
Source: PLoS One. 2025 Jan 22;20(1):e0313502. doi: 10.1371/journal.pone.0313502 (PMC11753638; doi:10.1371/journal.pone.0313502)
Supplement: Attachment — Submitted filename: response_letter.pdf [file pone.0313502.s003.pdf]

Response letter

Two experts in the field have carefully reviewed the manuscript entitled “What Emotions Does Music Express? Structure of Affect Terms in Music Using Iterative Crowdsourcing Paradigm”. Both the reviewers and I found the manuscript very interesting and relevant for music emotion research. However, one of the reviewers has made observations that need to be addressed.

In light of these reviews and my own reading of the manuscript, I am requesting a major revision and resubmission, in which you will need to respond to each point made in the reviews.
Let me focus on some points that the reviewers and I would like to see addressed. These are:

1) The rationale for the methodology, in particular the motivation, strengths and weaknesses of selecting expressed emotions by their association with types of music-related activities.

R: Thanks for the suggestion. We agree that we did not give sufficient background and justification to the task of nominating the expressed emotions and associating them with the music-related activities. We now have covered this in a better way in several sections of the manuscript, the introduction (p. 2), under “iterative paradigm” (p. 3) and the general discussion (p. 16). More specifically, we acknowledge that associating the terms with activities attempts to broaden the context of what kind of emotions music might be expressing as different activities require different emotional characters (preparing for an exercise has different expressive demands than trying to sleep or relax). We align these ideas with functions of music (Sloboda et al., 2009) and everyday uses of music (e.g., Randall & Rickard, 2017; Juslin et al, 2008).

2) Improving the quality of the writing in English.
R: Fair point and we recruited the services of a professional proof-reader to go over the text, leading to numerous language improvements.

3) Some minor points from me:
Line 328, “5129 unique tracks that added up to 5129”, is it correct?
R: This was poorly articulated although correct as participants in Experiment 2 submitted 5,129 unique tracks that exceeded the unique tracks (4,472) submitted by the participants in Experiment 1. But we agree that this reads awkwardly so we have altered the sentence to be clear.

Experiment 3, line 445: you say that the aim is to validate the Optimal model, but you use the factors from the Manual model instead. This is confusing for the reader.

R: This is a great point and we clearly have not explained our rationale sufficiently clearly to the reader. Our aim is to validate the *Optimal* model but we collect data on a broader set of terms (i.e., *Manual* model) as it is better to have a larger set of items to avoid misspecification of the factor structure (Brown, 2015) and most EFA and CFA cycles recommend starting broad rather than narrow (Fabrigar, Wegener, MacCallum, & Strahan, 1999, p. 278) as underfactoring (having fewer factors) is much more damaging for the CFA than overfactoring (starting with additional factors) (MacCallum, Widaman, Zhang & Hong, 1999, p. 96). We have now clarified our rationale of collecting a wider set of items (*Manual* model) to assess the fit of the *Optimal* model in the beginning of the Experiment 3.

References

Brown, T. A. (2015). *Confirmatory factor analysis for applied research*. Guilford publications.

Fabrigar, L. R., Wegener, D. T., MacCallum, R. C., & Strahan, E. J. (1999). Evaluating the use of exploratory factor analysis in psychological research. *Psychological methods, 4(3)*, 272-299.

MacCallum, R. C., Widaman, K. F., Zhang, S., & Hong, S. (1999). Sample size in factor analysis.. *Psychological Methods, 4(1)*, 84-99.

Lines 519-522: the association with the four Valence-Arousal quadrants seems problematic, specially association Factors II and IV with negative valence emotions.
R: That’s a great point and we were trying to be careful of not equating the Meta model with four quadrants as we only mentioned that the factors “...loosely resemble the four quadrants”. But you are completely right, it was stretching the similarity too much and we have dismantled and downscaled this association by characterising it more accurately (...”loosely resemble 2 out of 4 quadrants…”) and now describe the similarities (Factor I = Q1 and Factor III = Q3) and expose and discuss the discrepancies more carefully.

Reviewers' comments:

Reviewer's Responses to Questions

Comments to the Author

1. Is the manuscript technically sound, and do the data support the conclusions?

The manuscript must describe a technically sound piece of scientific research with data that supports the conclusions. Experiments must have been conducted rigorously, with appropriate controls, replication, and sample sizes. The conclusions must be drawn appropriately based on the data presented.
Reviewer #1: Yes
Reviewer #2: Yes

---

2. Has the statistical analysis been performed appropriately and rigorously?
Reviewer #1: Yes
Reviewer #2: I Don't Know

---

3. Have the authors made all data underlying the findings in their manuscript fully available?

The PLOS Data policy requires authors to make all data underlying the findings described in their manuscript fully available without restriction, with rare exception (please refer to the Data Availability Statement in the manuscript PDF file). The data should be provided as part of the manuscript or its supporting information, or deposited to a public repository. For example, in addition to summary statistics, the data points behind means, medians and variance measures should be available. If there are restrictions on publicly sharing data—e.g. participant privacy or use of data from a third party—those must be specified.
Reviewer #1: Yes
Reviewer #2: Yes

---

4. Is the manuscript presented in an intelligible fashion and written in standard English?

PLOS ONE does not copyedit accepted manuscripts, so the language in submitted articles must be clear, correct, and unambiguous. Any typographical or grammatical errors should be corrected at revision, so please note any specific errors here.
Reviewer #1: Yes
Reviewer #2: No

---

5. Review Comments to the Author

Please use the space provided to explain your answers to the questions above. You may also include additional comments for the author, including concerns about dual publication, research ethics, or publication ethics. (Please upload your review as an attachment if it exceeds 20,000 characters)

Reviewer #1: The paper presents an original research about the emotions expressed by music, an area underexplored in the literature of psychology of music. The main contribution of this paper is the design of an iterative method that aims at overcoming previous setbacks on experimental research in the field. The paper is clearly and consistently written. Detailed information about arguments and hypotheses, method, and data analyses is provided. Conclusions and implications are fully supported by the results. The experiments were conducted rigorously. Criteria about all the decisions made along both the experimental phase and the analyses of results were explained in detail. Supporting information is provided and protocols and materials are also available for replication in public repositories.

R: Thanks for the positive and reaffirming comments.

Reviewer #2: This is a very interesting and rigorously presented research study on emotions associated with music. The statistical analyses are well documented and seem to be done properly, but I am no expert so I cannot confirm this with full confidence.

My main concerns and request for revision are the following:

Abstract: you state that the structure is not well aligned with existing models, so what is does not relate to, but you do not say what it consists of instead. This does not yet provide a positive and clear statement of the novel contribution.

R: Thanks for highlighting this. We have revised the abstract to articulate more clearly the positives and how the structure of expressed emotions offers new terms and choices of structures that have not previously been available (from highly reduced to more granular and including concepts (“romantic”, “free”) and terms that have featured rarely in the literature (e.g. “dreamy”, “festive” and “in love”).

1) The methodology employed in the research is quite different from previous work that aimed at finding the main emotion terms that can be associated with music. It is not clear how deliberate this is. Are the authors arguing that this way of identifying emotion terms is more valid than previous surveys? If so, this should be part of the discussion of previous approaches in the literature review and introduction. Or was the study originally conceived to serve a different purpose (more about the emotional functions of music in everyday life) and was (part of) the data collected used for the purpose of identifying expressed emotions in music? If so, this should be acknowledged more openly.

R: Good point, and while the data collection clearly serves multiple purposes (what are the expressive terms but also for what are these terms used for in musical context referring to activities), the wider motivation was to be able to link participants choices of terms and tracks and check whether the terms actually communicate the intended emotions, and the possibility of connecting actual musical examples to the emotions expressed by them. In the past, the methods have focussed on the terms (what are the terms music can express in Juslin and Laukka, or in music induced emotion, how relevant are a long list of emotional terms for a sample of participants). We argue that these past efforts have limited utility because (1) they don’t actively widen the range of expressions/emotions considered which we do with activities/uses, and (2) they are regularly done without a reference to actual music, and (3) the choice of the most usable terms from a large possibility of term vocabulary can be more effectively refined when the terms are dynamically tested by their ability to communicate the intended expression across people. We did not want to antagonise the past research that in our view has collected emotion term data without any contextualisation and music (Zentner et al., 2008; Schindler et al., 2017; Juslin and Laukka, 2003) even though these studies have recognised these issues years after the studies and structures have been completed (Juslin et al., 2022; Strauss et al., 2024). We have now more explicitly outlined the multiple purposes of the dataset created and justified the role of functions as a useful way of covering the large and multifaceted expressive space of music.

2) Related to point 1, I feel there should be a more transparent discussion of the strengths and weaknesses of the approach taken in this study in the discussion. The way these terms were chosen, are they strictly speaking about what emotions music can express or are they indicating the affects and emotions that music can afford to listeners in certain circumstances? Such a relational perspective can be seen as a strength of the approach. It is about what music affords to listeners rather than purely about the potential communicative power of music and its characteristics. The role of lyrics in this sample of music should also be acknowledged.

R: This is a great suggestion, thank you. We appreciate the encouragement to discuss the strengths and weaknesses of the chosen approach more fully. In terms of the emotions expressed purely without the context and the emotions expressed in situations, context and activities, we think there is a limited value in pure expressive intention as most music is listened to and designed to be relevant for specific functions and contexts, although we could have gone much further in this by creating multiple scenarios where these terms might be relevant if we had attempted to fully embrace the context-sensitive of expressive purposes. But our approach is at least a minimal way of broadening the scope of expression, and even if we don’t fully situate and contextualise every decision to a real context, we nevertheless broaden the notion of what music

might express by reminding the participant that there are a number of common activities done concurrently with music. In our view this is already better than asking participants in a single context (e.g., a classroom for Exp. 1-2 or in a music festival for Exp. 3 or at home for Exp. 4 in Zentner et al., 2008) or without a context (Juslin & Laukka, 2003). Thank you for reminding us about the importance of lyrics, which is often absent from music and emotion studies. We agree that spelling out the relational perspective is a useful way of articulating the purpose of the design and we have now included this in the discussion (and we do refer to some of the enactive work on this and acknowledge the lyrics).

3) More generally what are the limitations that the approach taken may have promoted? The terms are chosen first of all in relation to activities that they can be associated with, and only quite a small number of activities are included. For example, mediation, sleep or connecting spiritually are not included, nor are having sex or preparing for war. A larger range of activities could have shown a larger range of expressions?

R: Thanks for raising this issue and we agree that this was now a minimal contextualisation, and more could have been done to expand the range of expressions. However, wider contextualisation might best be done properly with different scenarios to invoke the many situations and contexts appropriately. As we cannot rerun the experiment, the issue of context is now something that we have mentioned in the General Discussion, and we acknowledge that the context was limited and a broader set of activities would probably have enriched the vocabulary and widened the scope of the emerging structures. It would be worthwhile to explore the impact of the context more fully in future empirical work.

4) The literature review is not exhaustive. Whilst that may not be the purpose of the literature review in this case, the writing does suggest as if all relevant most recent literature has been included. However that is not the case – the below is for example also a relevant article. Also, did not GEMS also include expressed emotion? The 11 terms used in Laukka et al. cross-cultural study (2013, Emotion) seems also relevant? Schubert, E. (2003). Update of the Hevner adjective checklist. Perceptual and motor skills, 96(3_suppl), 1117-1122.
R: Thanks for these pointers and you are right, Schubert's study (2003) needs to be – and has now been – acknowledged but the study by Laukka et al., (2013) is a test of 11 eclectically chosen emotional expressions (some basic emotions and some aesthetic emotions) that does not really answer the same questions (what terms, what structures). Zentner's study (2008) did include emotional expression as part of the Experiment 2 but the paper only reports differences between felt and perceived emotions across genres, and unfortunately not the kind of outcomes that would have

enabled us or others to deduce which expressions are relevant or what is the likely structure within them.

This has been added to the introduction: "... An update to the taxonomies of emotion terms used since the 1930's [21] and 1950's [22] was proposed that consisted of 46 terms [23]". And few lines later: "Since the two surveys conducted in Sweden [6,7], the update by Schubert [23] of the adjectives describing music defined by Hevner [21] and Farnsworth [22], … "

5) A discussion of the terms is important too – affect terms, emotions, or even more broadly? The introduction is formulated in a way that suggests that this research will give an updated and more definitive list of emotions terms expressed by music. These promises should be checked with what the study actually offers in the end including in light of its limitations.

R: Good point and we on purpose keep the focus on affect terms and shy away from broader discussions of emotions as this would be more appropriate for the study of induced emotions. But this is nevertheless an important issue to raise and we think we deliver the promises mentioned in the aims and offer a dynamic list of appropriate affect terms (from 213 terms to 88 affect clusters to 21, 14, to 7 or 4 factors). We perhaps undervalue our findings as we don't have a strong recommendation for future studies to use a specific level of granularity of the obtained affect term lists or factors, but we are aware that these type of linguistic mappings to expressed emotions tend to vary across time (some terms from Farnsworth's work in 1950s such as "soaring", "heavy" or "sensational" was already dropped out of the 2003 evaluation by Schubert) and these are also language-specific. We now discuss this issue in the General Conclusions.

6) Abstract also needs to be updated in light of these comments.

R: We agree and we have rewritten most of the abstract now.

5) The English is not yet good enough. There are a lot of awkward sentences with poor grammar, which makes the reading challenging and reduces the clarity of what is written.

R: This is a fair criticism as long as it is about clarity and not stylistic requirement. We have now had an expert proof-reader to go through the manuscript, who addressed numerous cases where the writing style, clarity and grammar needed and could be improved.

Minor comments:
P2/30 l42. ‘inferior model’. This seems a bit strongly formulated. Also other models? How many, which do you mean?

R: Well spotted, and we agree that this does fit the style. This refers to a specific mapping exercise of affective cues in music by Cespedes-Guevara & Eerola (2018). While these authors indeed had polemic statements in their study, we adopt a more cautious language now, and describe the issues and models in more detail.

New version
“It is worth noting that the mapping between emotion cues in music and basic emotions tends to yield less consistent picture than when the cues are mapped to the quadrants in the affective circumplex”

P3/30 l71. Some missing references such as to Hevner and Schubert.
R: Thanks, Schubert and Hevner have been added to citations concerning affective circumplex.

Figure 1 legend – Level a refers to the input ‘of’ or input ‘to’? I think it should be ‘to’.
R: Thanks, we fixed it.

L127/8. Rather abstract first sentence under Rationale. Please improve.
R: Rewritten and shorter.

L141. You regularly state ‘aim’, which makes it confusing what the aim of the research is.
R: We agree, these reminders are unnecessary and confusing and have been removed from the other places (methods, materials) and now can only be found in the opening sentences of each experiment.

L150. Why is it important to collect this contextual information. This is buried in the methodology and needs more explicit discussion (see general comments).
R: This is a great point and we agree that we really did not motivate and introduce this properly. Now we have covered the purpose of using the activities as the minimal way of invoking the context of music listening in the introduction, and we have also revised the explanation of the iterative paradigm to reflect this reframing.

L162. Limited working proficiency? What is meant by that? Are you talking about English language proficiency?

R: Oops, the word English had dropped out, that has been fixed now (the sentence has been rewritten).

L169. Western music and classical music -> clarify this further, be more precise.
R: Good point, this was an attempt to summarise a complex distribution, which is given in full in SI Table 2 for all genres. We have revised the summary to be more accurate (at least list the top 5 genres) and emphasise the link to the SI Table 2.

L171. You present various statistics here but have not yet explained that you would be collecting those. Add a signposting explanation before summarising the data.
R: Thanks for the helpful pointer, we have added a sentence to the end of the materials section stating what demographic information we will be collecting and how (with what measures).

L225. Low ability to associate tracks – could it be that some search terms are less successful in retrieving an appropriate track, and could this be a confounding factor?
R: Possibly yes but this effect is probably very, very small. Participant's ability to associate tracks with the affect terms may be influenced by their ability to complete the task but we do not have information about their individual abilities. However, the potential variability among the participant's ability is likely to be distributed randomly across the data as each term received at least 50 decisions from 46-50 different participants. For this reason it is unlikely that a specific term would have suffered from a particular individual or individuals having significantly lower ability to do the task. In other words, the potential error source (low ability) is randomly distributed in this data. This is still a valid point and could be explored in future.

L243 Silhouette scores? More info is needed.
R: We clearly had omitted essential information about the data reduction and thanks for alerting us to this issue. We have rewritten this section and described the k-means clustering operation in full and provided the steps done and the supporting references for the specific techniques such as choosing the number of clustering with silhouette method (p. 7).

L247 not clear what analysis was done to reduce to 88 clusters. There were 9 activities, so how did those play a role in the reduction as suggested above?
R: We now have documented the k-means clustering operation in full and provided the steps of the analysis. The clustering was done from the term-activity co-occurrence matrix (213 x 9) that was subjected to k-means clustering. The number of clusters taken forward in this analysis was determined by the silhouette analysis that showed the peak at 88 clusters.

Figure 2. Panel A, B and C are not matched in order with the order presented in the figure caption.
R: This error has now been fixed and the figure caption has been rewritten.

L264-5. Check grammar
R: Thanks, now rewritten and the plot has been redrawn to illustrate the distribution of the terms in the affective space better by adding marginal plots.

L282 and elsewhere. Check what tense you use where. In Exp 2, method, you used past tense. Here you use present. (aim was / aim is).
R: Thanks, now fixed (switched to past tense) and adjusted in two other sections.

L302. do you mean that participants did two times an activity association task and four times an annotation subtask? They could not do as many tasks anymore as they wanted? The way this is phrased is unclear as it can also be two different activity association tasks.
R: You are right, this was unclear. Now the section is explained in the same manner as in Exp. 1 where the different number of subtasks is now specified“... Each participant did a minimum of 7 tasks (2 activity associations, 1 track submission, and 4 track annotations) which were presented in a random order.”

L307 and the rest of that section. This is data analysis, not data collection procedure.
R: Fixed.

L314. This procedure (division) is that to get a proportion? Can be further explained.
R: Yes, otherwise the numbers would reflect the raw counts and we wanted to have proportions that also reflect the communication accuracy of the term through the track submission and annotation subtasks. We have now clarified the text.

L322. Why binary terms and why 22. I am lost a bit here without further explanation.
R: You are right, the sentence did not explain what is the logic behind 22 and 66. The reason for the 22 terms is that the participants were only exposed to randomly chosen 22 terms in the interface to make the task plausible while the whole response array has 88 terms. For this reason we have binary data (chosen, not chosen) for 22 terms and NAs for the rest of the 66 terms, which were randomly selected. For this reason, each row is unique combination of these decision for a total of 28,550 rows. We have rewritten this section to be clearer.

L330. Not majority of the track, but the largest number of tracks?
R: Thanks, changed.

L333. The median of 211 activities associations were obtained. This is phrased in a rather confusing manner. Please rephrase and clarify. Also, 211 unique associations or are just reporting how many responses? I assume the latter. But that is not so relevant, is it?
R: True, this was not clearly written and has now been revised. It is indeed the latter (median of 211 responses of activities for each term). We thought of reporting the descriptive numbers before moving onto the analysis proper, and we think these contextualised indices are more meaningful than saying 45,212 decisions were made by 2508 participants.

L373. ‘separated into two’. Do you mean each was split into to two to yield 10, or combined into two factors?
R: We meant that each factor was split into two (positive and negative version) as you surmised and have amended the text accordingly.

L374. by considering computing? Or do you mean by computing? Surely you do not just consider it?
R: Yes, this was changed to active term (“assessing the increase”).

L375. were attempted to combine is not good grammar
R: True, the sentence has been rewritten.

L421. In General Discussion. ‘the’ is missing.
R: Fixed.

L433. Did participants clearly chose music to express this? I would discuss and unpick this a bit further (see general comments).
R: This was expressed too strongly here and we dropped this direct claim.

L443. Further data – as will be done next.
R: Good, thanks, added.

L445-7. First sentence of the section is hard to parse. Please improve.
R: You are right. We rewrote this part of the discussion and focus now more on the term good and how that could be interpreted (good music, or feeling good).

L479-480. check sentence the factors are collected in this experiment, are not they? Or perhaps 'with' is not the correct word?
R: The sentence required rewriting and now it expresses the intention better.

Table 3. table heading. not clear what is being correlated as you say 2x 'between' in this sentence.
R: This was unclear. We have rewritten the caption to be explicit:

“Correlations between affect terms in Experiment 3 and emotion concepts from the past studies, where Exp. 2 refers to the same terms in the track annotation task in Experiment 2 of the present study (N = 4780), Set 600 provide ratings of three affect dimensions (N = 419), and Soundtracks offer ratings of five discrete emotions (N = 110)

Table 3 and elsewhere: check when you do and do not include a 0 before the full stop. You say p < .01 but also r = 0.01. All numbers that range between 0 and 1 should be treated the same, so this would need to be r = .01 to be consistent. (and making the r and p italic).
R: Thanks, all p values and correlations now reported consistently without leading zeros.

L497. Dreamy is ‘simply’. Why simply and how can you be so sure?
R: Oops, that did not make sense. We just described the correlations between the known concepts (valence, arousal) and some of the concepts, so there is nothing deeper here. The description is now very basic and factual.

L499. what do you mean by 'the more dimensional concepts?
R: This was poorly phrased and thanks for calling it out. We simply meant correlations between the affect terms and dimensions are higher on average than between basic emotions and affect terms. We have now rewritten this and supplied the mean correlations for both comparisons (r = .36 for dimensions, r = .27 for basic emotions) to qualify the observation.

L502. and inspired and sensual are themselves the most poorly defined?
R: True, and now this has been explicitly mentioned in the manuscript.

L528 and elsewhere. affect terms or concepts? You switched terminology. Be consistent for clarity. Or is this change on purpose?
R: You are mostly right, and we keep referring to affect terms but we also introduced “concepts” in Exp. 3 when we talked about valence and arousal and basic emotions

from the past studies. However, we were not always consistent as there were several places where we used concepts for terms wrongly, and now we have made sure that we only use concepts for arousal/tension/valence and basic emotions and when we refer to the affect terms, we always refer to terms/affect terms.

L534. acknowledge that quadrant is close to .9
R: Good pointer, this has now been added.

L605. different sets of structures -> is this undermining the confidence in the outcomes too much? Formulate less strongly?
R: Excellent point, and it is likely that other methods would create alternatives of the structures that would be relatively minor. We have softened the statement now.

L625. ‘less descriptive’ should this be ‘fewer descriptive’?
R: Thanks, fixed.

S1 Table 1. Why this order of continents? It is not alphabetical nor in order of number of participants.
R: True, the continents have now been arranged in order of the number of participants.

S1 Table 4. How can the rank be alphabetical?
R: Thanks for identifying this crucial omission, the table was missing the actual frequency rank column, which now has been added in.
