# Supplementary material for: What emotions does music express? Structure of affect terms in music using iterative crowdsourcing paradigm
Source: PLoS One. 2025 Jan 22;20(1):e0313502. doi: 10.1371/journal.pone.0313502 (PMC11753638; doi:10.1371/journal.pone.0313502)
Supplement: Attachment — Submitted filename: Response letter_R2.docx [file pone.0313502.s004.docx]

**Response letter**

Reviewers' comments:

Reviewer #2: My only remaining request is to improve the explanation of the rationale for the study in the abstract. Is it really the case that we know more about the structure of felt emotion than expressed emotion and has that really been more investigated? Such comparisons are hard to make. I would instead explain that expressed emotion in music is typically investigated without looking at the contexts in which music is experienced, which may lead to the uncovering of reduced and abstracted beliefs about expressed emotion rather than closely represented experiences of emotions expressed (also should it be affect instead?).

R: Thanks for this helpful suggestion that clarifies the rationale and contextualises the study much better. Although we do think that felt emotions have received more attention in terms of the emotion structures, but we completely agree that this is kind of beside the point made here; It is much more valuable to say that the context has not been looked at, and this might have simplified/reduced the range of appropriate affects and structures. We have now rewritten the abstract to reflect this crucial shift in the emphasis, and this really helps to portray the present study much better than the previous version. We had to shorten other parts of the abstract, but now it is 200 words and communicates the novel aspects of the study much better. Thank you!

In this revision, we also incorporated an observation from a colleague after a conference presentation of this study. The observation relates to the interpretation of the meta-factors in Experiment 3, which bear some striking resemblance to the broad music preference factors (5 factors in the MUSIC model by Rentrow et al., 2011). We point this semblance out and ponder whether the possible underlying reason for the similarity is the contextualised nature of the work done here and how music genres are often also used by listeners. We hope this uncontroversial short addition (108 words, see page 15 of the manuscript) to the interpretations could also stimulate future work on affects and uses of music.
